# Projecting Overwintering Regions of the Beet Armyworm, *Spodoptera exigua* in China using the CLIMEX Model

**DOI:** 10.1673/031.012.1301

**Published:** 2012-02-01

**Authors:** Xia-Lin Zheng, Pan Wang, Wen-Jie Cheng, Xiao-Ping Wang, Chao-Liang Lei

**Affiliations:** Hubei Insect Resources Utilization and Sustainable Pest Management Key Laboratory, College of Plant Science and Technology, Huazhong Agricultural University, Wuhan 430070, China

**Keywords:** geographical distribution, projection

## Abstract

The beet armyworm, *Spodoptera exigua* Hübner (Lepidoptera: Noctuidae) is a serious agricultural pest worldwide. However, population sources of *S. exigua* in outbreak regions are still vague due to the lack of understanding the distribution of overwintering regions, especially in China. In the present study, the potential overwintering regions of *S. exigua* in China are projected using the method of Compare Location in the CLIMEX model in order to understand the population sources in outbreak regions and establish an accurate forecasting system. The results showed the southern and northern overwintering boundaries near the Tropic of Cancer (about 23.5 ^°^N) and the Yangtze River valley (about 30 ^°^N), respectively. Meanwhile, the projection was supported by the data of fieldwork in 14 countries/cities during winter from 2008–2010. In conclusion, results of this study indicated that the overwintering regions of *S. exigua* were accurately projected by the CLIMEX model.

## Introduction

In response to cold environmental conditions, insects have developed overwintering adaptations that enable them to escape temporally through dormancy (Tauber et al. 1986). However, the dormant stages and regions of an insect are diverse at different latitudes attributed to thermal constraints (Alexander 1968). Therefore, understanding dormant stages and regions of insect in the field, particularly the most serious agricultural insect pests, will facilitate understanding local population sources, establishing an accurate forecasting system and drawing up effective control strategies in time (Bale and Hayward 2010).

The beet armyworm, *Spodoptera exigua* Hübner (Lepidoptera: Noctuidae), is a persistent agricultural pest in many areas of the world (Brady and Ganyard 1972). *Spodoptera exigua* has no known diapause (Fye and Carranza 1973) and can over—winter in the pupal stage at San Diego, Orange, and Ventura Counties of California (Trumble and Baker 1984); the country of Jordan (Al-Abbadi 2001); Bayramaly in Turkmenistan (Kurdov 1986, 1987); Dali State of Yunnan Province (Li 2005) and Hengyang City of Hunan Province (Yin et al. 1994) of China, and in the larval stage at Kagoshima Prefecture of Japan (Suenaga and Tanaka 2000). However, there are different views regarding whether *S. exigua* can over—winter in some regions in China. For example, some considered outbreak populations in Shandong Province migratory from southern provinces (Feng et al. 1995), while others insisted that they originated from the local overwintering population (Wang et al. 2002). Similar debates were also raised concerning population sources in Jiangsu Province (Han et al. 2003, 2004, 2005; Wang et al. 2007), Henan Province (Wu et al. 2000; Guo et al. 2005), and the Yangtze River valley (Yin et al. 1994; Lu and Xie 1995).

The direct evidence about overwintering of *S. exigua* can only be obtained by finding surviving individuals in the field during winter. However, the exact overwintering regions of *S. exigua* in China are unclear, because correlative historical data are so limited that understanding the overwintering regions remains a considerable challenge. Therefore, appropriate data management software is a good alternative choice to predict the potential overwintering regions of *S. exigua* in order to provide a theoretical map to guide fieldwork during winter.

CLIMEX (Hearne Scientific Software, www.hearne.com.au) has been proven to be a reliable inferential model (Kriticos and Randall 2001). The model is based on the assumption that if the regions where species live are ascertained, the potential geographical distribution in relation to climate can be estimated. So far, it has been widely used to determine the potential geographical distribution of poikilothermal organisms, such as *Culex gelidus* (Williams et al. 2005) and *Harmonia axyridis* (Poutsma et al. 2008). In addition, this model assumes that species at a given location experience one season that is favorable for population growth, and one that is unfavorable (Sutherst et al. 2004). These are referred to as growth or survival and stress seasons, respectively. While the occurrence during winter may be limited by either temperature or other factors, the Ecoclimatic Index (EI) is usually related to the insect growth responding to climate, and can be used to describe the population dynamic.

This study is an attempt to project the potential overwintering regions of *S. exigua* in China by using CLIMEX model based on (i) physiological requirements, (ii) the recorded overwintering regions of this species in the world, and (iii) meteorological data from 758 stations in China.

## Materials and Methods

### The CLIMEX model

CLIMEX model (version 2.0) was used in this study. CLFMEX was used to predict the potential geographical distribution of an organism combined with meteorological data through a series of annual indices (Sutherst et al. 2004). It operates in two modes: Compare Locations and Compare Years. In the former, it enables the user to predict the potential geographical distribution of a species or a pair of species in relation to climate based on its climatic preferences, while in the latter the response to climates in different years at the same place is compared. In CLFMEX 2597 locations (meteorological stations) worldwide are included, of which 86 locations are recorded in China. The climatic data associated with these locations span the period 1960–1990.

Species parameters inferred from literature and experimental values are part of the input in the CLFMEX model, and describe the modeled organism response to climatic data. In CLFMEX, an annual Growth Index describes the potential of growth for a population during the favorable season. There are four stress indices (cold, hot, wet, and dry), and in some cases the interactions between them (cold—wet, cold—dry, heat—wet, and heat—dry) describe the extent to which the population is reduced during the unfavorable season. The growth and stress indices are combined into an EI, which is scaled from 0 to 100 to give an overall measure of climatic suitability for the species concerned. Generally, an EI > 0 indicates that the location is possible for species growth, and an EI of more than 30 represents a very favorable climate for a species (Sutherst et al. 2004). In the current study, values EI > 30 are considered more suitable for *S. exigua.*

In this study, four steps for projecting potential overwintering regions of *S. exigua* by CLIMEX are provided as follows:

(i) All original literature and databases concerning the occurrence and overwintering regions of *S. exigua* in the world were collected. Then, biological parameters were entered to obtain one map (geographical distributions). The parameters, including temperature and moisture, could be adjusted to match with the known distribution introduced in literature or databases if the distribution in one region was out of actual range.
(ii) On the basis of the above result, four parameters measuring the tolerance of properties of the species were set. These were the cold stress temperature threshold (TTCS), cold stress accumulation rate (THCS), temperature threshold (TTHS), and heat stress accumulation rate (THHS), which were adjusted to obtaining another map, in which the overwintering regions were consistent with the literature and databases.
(iii) A new meteorological database and location file of China was created, with the .loc/.met format, including a total of 758 stations. The detailed process of importing the new MetManager database file into CLFMEX is described in the User's Guide (Sutherst et al. 2004). Then, the potential overwintering regions of *S. exigua* in China were projected by the method of Compare Locations based on the parameters.
(iv) The final EI for *S. exigua* in China was generated, and the output files were integrated into Arc-GIS 9.3. Tonnang et al. (2010) adopted the method of Kriging within Arc-GIS 9.3 to obtain the EI surfaces. The method provided a visual description showing species ranges rather than simple points, particularly for poorly sampled regions (Anderson et al. 2002; Tonnang et al. 2010).

#### Known distribution of *Spodoptera exigua*

*Spodoptera exigua* shows a cosmopolitan distribution, which was determined from extensive literature, various websites, and a survey of locality data held by various institutions. These records indicate that *S. exigua* shows a relatively broad distribution between latitudes 45 ^°^S and 64 ^°^N (CAB 1972).

#### Fitting CLIMEX parameters

Stress parameters were used to calculate the stress indices and were defined by a threshold and a weekly accumulation rate. A detailed presentation of stress calculations was available in the CLFMEX User's Guide (Sutherst et al. 2004). Stress indices, which scale between 0 and infinity, were manually adjusted so that stresses would largely constrain the population from expanding beyond its present observed distribution limits in the world. The parameter values used to project the distribution of *S. exigua* are given in Table 1.

#### Temperature index

A value of 15.06 ^°^C was set as the minimum temperature for development (DV0), as experimental results indicated that development of all stages did not take place below this temperature (Han et al. 2003). According to the experimental results, the lower (DV1), upper (DV2), and maximum (DV3) temperature threshold for population growth were set at 26, 29, and 36 ^°^C, respectively (Xu et al. 1999).

#### Moisture index

Suitable soil moisture conditions are necessary for *S. exigua* pupation. Experimental results showed that the soil moisture which larvae preferred ranges from 1–20%, which was suitable for larvae to pupate and grow rapidly (Fye 1978). Larval preferences decreased significantly when soil moisture was > 25% before pupation. Moreover, pupae could not survive in water (Ma et al. 2000). On the basis of experimental data, the soil moisture threshold (SM0), soil moisture values for optimum (SM1 and SM2) and upper (SM3) were set at 0.03, 0.31, 1.00, and 2.00, respectively.

#### Cold stress and thermal accumulation

Cold stress can be defined in three ways. Firstly, animals may die because the daily thermal accumulation is too low to maintain metabolism. Alternatively, a species may not survive if exposed to excessive low temperatures. As a third option, a species may spend the winter in locations where it is buffered from extreme minima and maxima (Sutherst et al. 2004). Previous research had shown that *S. exigua* is a freezing susceptible insect (Kim and Kim 1997), and it has never been observed to diapause (Fye and Carranza 1973). *Spodoptera exigua* could not endure persistent low temperature (Kim and Song 2000a), though it exhibited lower supercooling points of eggs, larvae, and pupae (Kim and Kim 1997; Kim and Song 2000b; Jiang et al. 2001; Han et al. 2005). Under natural conditions, low temperature was identified as one of the main limiting factors of *S. exigua* populations based on the knowledge of biology and tropical/temperate distribution of this species. According to the survival time at 5 ^°^C to -10 ^°^C (Jiang et al. 2001), the TTCS and THCS were set at 0.73 ^°^C and 0.1699 week^-1^.

In addition to lethal cold temperatures, the distribution of *S. exigua* in cool environments is likely to be restrained by the annual integral of temperature and time required to complete their life cycle (PDD). Following Han et al. (2003), the sum of degree—day on a 15.06 ^°^C (DV0) basis to complete one generation of *S. exigua* was set to 265.6 ^°^C days.

#### Heat stress

CLFMEX provides two heat stress models, the temperature threshold and the degree—day threshold. *Spodoptera exigua* exhibited strong ability to withstand high temperature conditions (Zhai et al. 2010). According to previous experimental data, the heat stress temperature threshold (TTHS) and the rate of heat stress accumulation (THHS) were set at 40 ^°^C and -0.0177 week-^1^.

#### Dry and wet stress

Dry stress accumulates when the soil moisture level falls below the dry stress threshold (SMDS). On the other hand, wet stress accumulates if the soil moisture level exceeds the wet stress threshold (SMWS). On the other hand, wet stress accumulates if the soil moisture level exceeds the wet stress threshold (SMWS). Information of SMDS, dry stress rate (HDS), SMWS, and wet stress rate (HWS) stress parameters were not found in the literature, so they were set iteratively in order to limit expansion where nothing is known about species occurrence (SMDS: 0.02; HDS: -0.005 week-1; SMWS: 2.5; HWS: 0.002 week-1).

#### Initial steps toward model validation

The goal of fieldwork was to validate the accuracy of the potential overwintering regions from the model. The southern and northern overwintering boundaries were located at the Tropic of Cancer (about 23.5 ^°^N) and the Yangtze River valley (about 30 ^°^N), depending on the results of CLIMEX model (see below). Thus, fieldwork during winter from 2008 to 2010 was chosen in Sanya (109.31°E, 18.14 ^°^N, investigation area was 90 m^2^), Guangzhou City (113.23 ^°^E, 23.16 °N, 300 m^2^), Longnan County (114.79 ^°^E, 24.91 °N, 200 m^2^), Nanchang City (115.89 °E, 28.68 ^°^N, 500 m^2^), Yongxiu County (115.82 °E, 29.04 ^°^N, 2000 m^2^), and the cities of Yibin (104.56 ^°^E, 29.77 °N, 3000 m^2^), Wuhan (114.3 °E, 30.5 ^°^N, 20000 m^2^), Jurong (119.16 °E, 31.95 ^°^N, 5000 m^2^), Nanjing (118.78 ^°^E, 32.04 °N, 3000 m^2^), Xi'an (108.95 °E, 34.27 ^°^N, 500 m^2^), Tai'an (117.13 °E, 36.18 ^°^N), Anqiu (119.2 °E, 36.42 °N), Zhangqiu (117.53 °E, 36.72 ^°^N), and Beijing (116.46 ^°^E, 39.92 ^°^N). The investigation areas of the last four were absent. The method of finding eggs, larvae, pupae, and adults was described in Wang et al. (2009) and Zheng et al. (2010).

### Results

#### The iteration process

**Figure 1.**
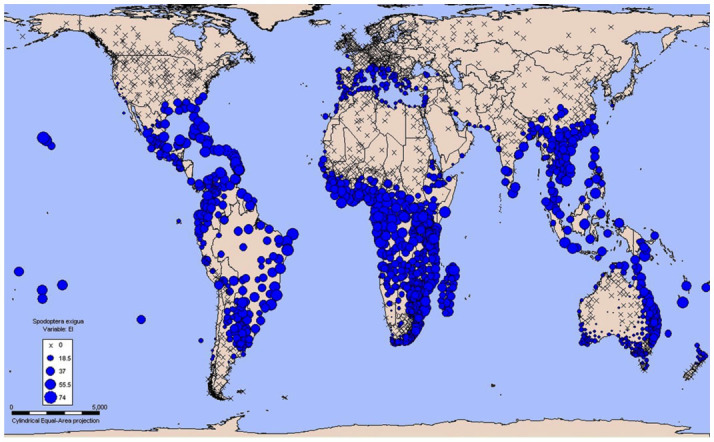
CLIMEX map of the world indicating Ecoclimatic Indices (El) of *Spodoptera exigua.* Crosses represent an El of zero. Spots represent El's greater than zero. The larger spots indicate that the climate at the station is more suitable for this species. High quality figures are available online.

Values of some parameters demonstrated large effects on the EI and therefore on the potential distribution during the iterative adjustments and comparison process for *S. exigua.* The results of original parameters indicated that *S. exigua* could overwinter in Washington, Virginia, and Arkansas of the United States, Greenwich of the United Kingdom, France, and Nagasaki of Japan. However, there were no actual observations of overwintering in these regions. Generally, temperature affects the distribution in the North-South direction, and moisture affects the distribution in the Coast-Inland direction (Hou and Zhang 2005). Therefore, we upgraded TTCS from 0.73 to 3.2 ^°^C, and downgraded THCS, THHS, and TTHS from -0.1699 to -0.20 week^-1, 0.0177 to 0.0163 week-1, and 40 to 36 °^C, respectively, to match its absence. Then the distribution of overwintering regions corresponded closely with the observed distribution of *S. exigua* (Figure 1).

**Table 1.**
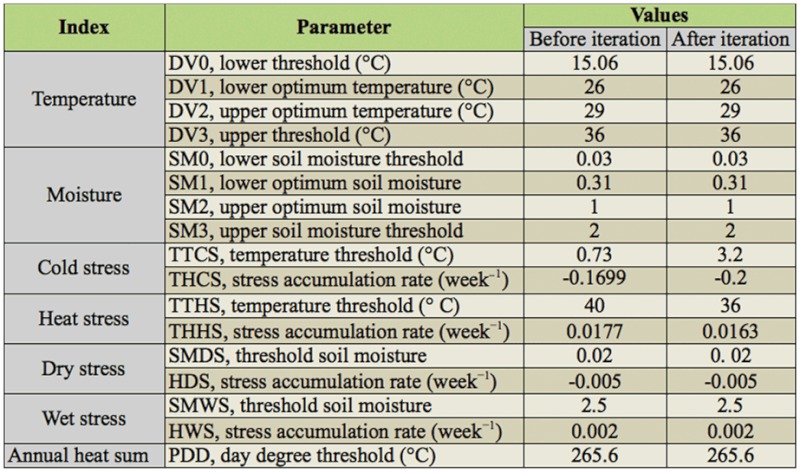
CLIMEX parameter values used for *Spodoptera exigua.*

**Figure 2.**
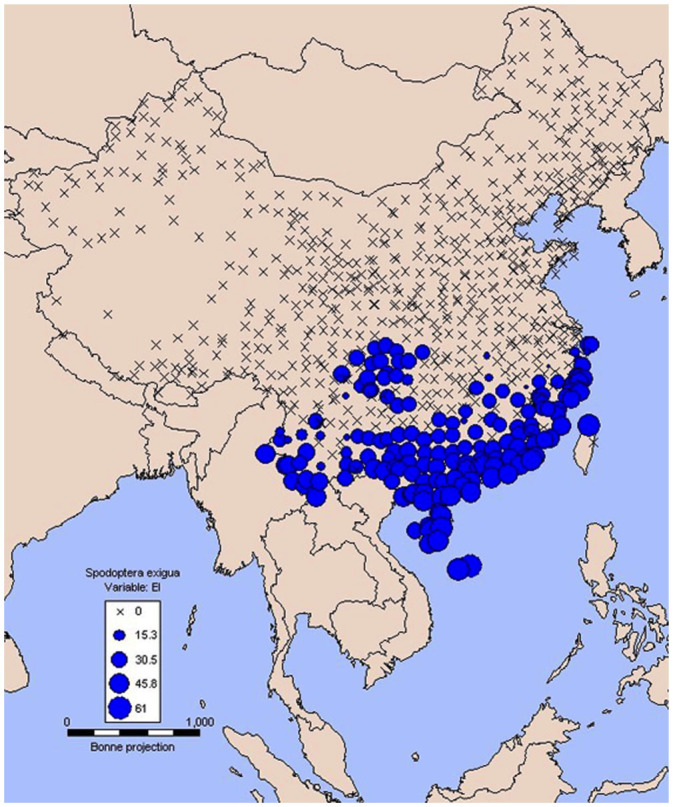
CLIMEX map of over—wintering regions of *Spodoptera exigua* in China inferred from its known world over—wintering sites after importing total of 758 meteorological stations. Crosses indicate unsuitable over—wintering locations. Spots represent El's greater than zero. The larger spots indicate that the climate at the station is more suitable for over—wintering of this species. High quality figures are available online.

**Figure 3.**
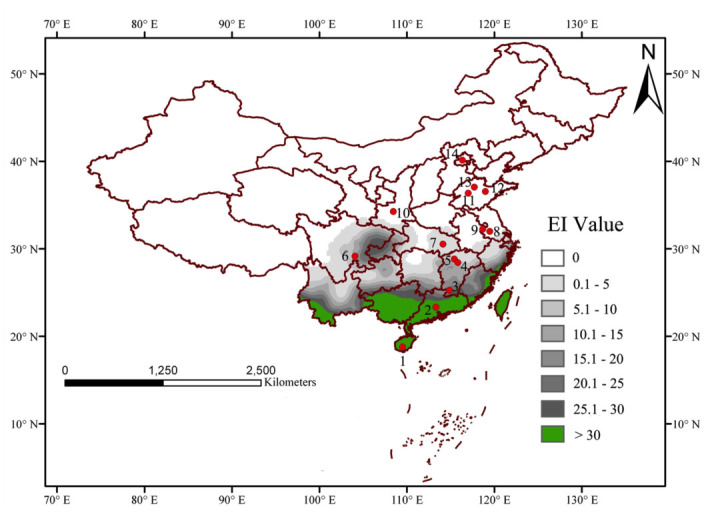
Over—wintering regions of *Spodoptera exigua* under current climate in China. The map was constructed by Arc-GIS using the Ecoclimatic Indices (El) obtained from CLIMEX parameters in Table I. In these areas covered color with green indicates perennial damage regions and covered with gray indicates over—wintering regions (gray shades indicated suitable areas according to the El values, and the darker the color is, the more suitable the area). Red dots represent the site of fieldwork during winter. 1, Sanya; 2, Guangzhou; 3, Longnan; 4, Nanchang; 5, Yongxiu; 6, Yibin; 7, Wuhan; 8, Jurong; 9, Nanjing; 10, Xi'an; 11, Tai'an; 12, Anqiu; 13, Zhangqiu; 14, Beijing. High quality figures are available online.

When these parameters were applied after iteration (Table 1) on the basis of meteorological data of 758 stations in China, the map of EI suggested that the southern and northern overwintering boundaries were located at the Tropic of Cancer (about 23.5 ^°^N) and the Yangtze River valley (about 30 ^°^N), respectively (Figures 2 and 3). Usually, the EI values range between 0 and 100. An EI value equal to 0 indicates that the location is unsuitable for long term survival of the species. In contrast, the larger EI value shows that the more suitable climate is at that station. Figure 3 shows an Arc-GIS map using the EI value of *S. exigua* under the current climate, and these areas covered with green indicate the perennial damage regions (EI > 30), which are consistent with previous studies. In the overwintering regions, gray shades represent suitable areas according to the EI values, and the darker the color is, the more suitable the area. White regions indicate locations unsuitable for *S. exigua* overwintering (Figure 3).

#### Match with current distribution of over—wintering

Previous studies have been reported that *S. exigua* could overwinter in the pupal or larval stage in many regions. Based on these historical data, the potential over—wintering regions in China were projected (Figures 2 and 3). Under current climate conditions, 12.8% of EI values were greater than 30. Previous research indicated that all developmental stages of this insect were found throughout the year at Taiwan, Hongkong (Chen et al. 1988; Lv et al. 1992), Shenzhen (Dai et al. 1999), and Xiamen (Fang et al. 1998) in China. Our projection was consistent with these historical data in this study. Thus, green areas in Figure 3 were defined as belonging to perennial damage regions. Meanwhile, the potential overwintering regions in China were illustrated as gray shaded areas (Figure 3) in which the southern and northern overwintering boundaries were located at the Tropic of Cancer (about 23.5 ^°^N) and the Yangtze River valley (about 30 ^°^N).

#### Initial steps toward model validation

First, all stages of this insect were found throughout the year in Sanya and Guangzhou Cities, so these regions can be considered as perennial damage regions. Furthermore, living larvae and pupae were found from January to March in overwintering regions projected by the CLIMEX model. For example, in Wuhan City of Hubei Province, living larvae were found on *Spinacia oleracea* (22 individuals) on 12 January 2009, and on *Brassica compestris* var. *purpurea* (1 individual) in March 2009. Living larvae were also found on *Raphanus sativus* (6 individuals) in Nanchang City, on *Brassica rapa pekinensis* (1 individual) in Yongxiu County, and on *Brassica oleracea* var. *capitata* (1 individual) in Longnan County of Jiangxi Province in January 2010. Once again, living pupae were found in the field on *Allium schoenoprasum* in Yibin City (1 individual) of Sichuan Province, and in the field on *Raphanus sativus* in Nanchang City (5 individuals). Furthermore, it is worthy of mentioning that adults were trapped using sex pheromones (NewCon Inc., www.newcon-inc.com) in Yibin City on 26 January (1 individual), 26 February (1 individual), 23 March (4 individuals), 29 March (1 individual), and 30 March 2010 (9 individuals). However, no individuals were found in Nanjing and Jurong cities of Jiangsu Province, Xi'an City of Shanxi Province, Tai'an, Zhangqiu and Anqiu cities of Shandong Province, or Beijing city. Therefore, we concluded that & *exigua* could overwinter in Yibin, Wuhan, Nanchang, Yongxiu, and Longnan cities/counties, and could not overwinter in Nanjing, Jurong, Xi'an, Tai'an, Zhangqiu, Anqiu, and Beijing cities.

### Discussion

Although others have used CLIMEX to predict the expansion and final range of insect species (Sutherst and Maywald 2005), we believe that this study is the first attempt to explicitly predict the overwintering range of an insect. Our results indicated that the southern and northern overwintering boundaries of *S. exigua* are located at the Tropic of Cancer (about 23.5 ^°^N) and the Yangtze River valley (about 30 ^°^N). Most importantly, field data positively supported the projection made in this study. The perennial damage regions of *S. exigua* (EI > 30) in the present study were consistent with that in previous research (Chen et al. 1988; Lv et al. 1992; Fang et al. 1998; Dai et al. 1999). Meanwhile, the potential overwintering regions (0 < EI < 30) of *S. exigua* in China were clearly demonstrated (Figure 3). Gray shades indicated suitable areas according to the EI values, and the darker the color was, the more suitable the area was. In 2001, various isotherms in China were divided into non-overwintering, possible overwintering, and perennial damage regions for *S. exigua* (Jiang et al. 2001). These results were similar with our model projection based on meteorological data. However, the northern overwintering boundary in our results was more southern than that of Jiang et al. (2001). Whether *S. exigua* could overwinter from approximately 30 ^°^N (Yangtze River valley, in this study) to 38 ^°^N (Jiang et al. 2001) requires further study.

CLFMEX was used to project the potential geographical distribution according to the response of an organism to meteorological data. However, the few meteorological stations (only 86 in China) recorded in CLFMEX may result in overestimation or underestimation for the overwintering regions. For instance, a few regions where *S. exigua* could overwinter probably were not covered when overwintering regions were projected only using the climatic stations and data contained in CLFMEX, such as Wuhan and Hengyang cities. Therefore, meteorological data of 758 stations (including 86 locations contained in CLEVLEX) in China were imported to ensure the accuracy of the projection.

Furthermore, overwintering regions of species projected by relating biological data generally tend to overestimate or underestimate the actual extent, especially using only the environmental conditions of the sites where the species have been recorded. As a matter of fact, factors that influence insect overwintering also involve light, humidity, rainfall, host plants, drought, natural enemies, and other indirect factors. On the one hand, experiments aimed at elucidating the effects of humidity, natural enemies, and pathogens on insect overwintering were so limited that we were unable to evaluate whether these factors may lead to higher or lower mortality during winter. Under natural conditions, single and multiple factors could influence the survival of overwintering insects. For example, entomopathogens could parasitize individuals and lead to high mortality of insects, especially in higher humidity (Riddick and Schaefer 2005; Harwood et al. 2006; Riddick 2006). Similarly, parasitoids could also decrease the survival of overwintering insects (Ceryngier and Hodek 1996). On the other hand, *S. exigua* pupated in the soil was observed to over—winter in many regions of the world (Trumble and Baker 1984; Kurdov 1986, 1987; Yin et al. 1994; Li 2005). Rainfall, especially after pupation but before moth emergence, can reduce the survival of pupae (pupating in the soil) by disrupting emergence tunnels (Murray and Zalucki 1990; Montoya et al. 2008). However, studies concerning the effect of rainfall on the survival of *S. exigua* pupae were rare. If these factors mentioned above were taken into consideration, the model projections would be altered. Hence, synergism in multiple factors in the field needed to be considered for more exact projections.

Although fieldwork provides the direct evidence to clarify the presumption or projection, we must acknowledge the difficulties doing field investigation during winter. In the present study, 14 sites from south to north were chosen for the fieldwork. Perhaps this would be considered inadequate for a comprehensive validation, so we recommend additional field investigations in the future to further test the model.

## Abbreviations:

El: Ecoclimatic Index;
SMDS: dry stress threshold;
SMWS: wet stress threshold;
TTCS: cold stress temperature threshold;
THCS: cold stress accumulation rate;
THHS: heat stress accumulation rate;
TTHS: temperature threshold
